# Supplementary material for: Diffusion in dense supercritical methane from quasi-elastic neutron scattering measurements
Source: Nat Commun. 2021 Mar 30;12:1958. doi: 10.1038/s41467-021-22182-4 (PMC8009954; doi:10.1038/s41467-021-22182-4)
Supplement: Supplementary file 1 — Supplementary Information [file 41467_2021_22182_MOESM1_ESM.pdf]

Supplementary Information

Diffusion in dense supercritical methane from quasi-elastic neutron scattering measurements

Ranieri, U. et al.

Supplementary Note 1: Rotational contribution to the measured spectra

The basic principle of time-of-flight QENS is that a monochromatic neutron beam is scattered by the diffusing nuclei in the sample and one measures the scattered intensity as a function of the scattering angle and neutron time of flight, which are then converted into momentum and energy transfers, $\hbar Q$ and E. For methane, because the incoherent cross-section of the proton is much larger than any other cross-section, the scattered intensity is dominated by the self-dynamic structure factor $S_{\mathrm{inc}}(Q, E)$ of the protons. The usual approximation is to consider that the different kinds of movements (vibrations, translations, and rotations) are uncoupled, which is essential to derive an analytical expression for $S_{\mathrm{inc}}(Q, E)$. For each of them, the timescale of the motion has to be compared to the timescale window probed by the instrument, the upper limit of which is set by the energy resolution and the lower limit by the accessible energy transfer range. The elastic instrumental energy resolution was about 0.08 meV during our experiment on IN6-SHARP, so quasi-elastic lines down to 0.1 meV in width could be analysed, corresponding to a characteristic time of 13 ps (the characteristic time is defined as $\hbar/\Delta E$, with ΔE the half width). The maximum energy transfer values available on the Stokes side were 1-2 meV, corresponding to about 1 ps. On the anti-Stokes side, the minimum energy transfer values varied from –1.25 meV at Q=0.4 Å^{-1} to –16 meV at Q=1.9 Å^{-1}, so in principle, timescales as small as $\sim$0.08 ps could be accessed at high Q.

The contribution of the vibrational motion (high-frequency motion around the mean transla-

tion path or intramolecular vibration) can be approximated with a factor $\exp(-Q^2\langle u^2\rangle/3)$, where $\langle u^2\rangle$ is the mean square vibrational amplitude. This is the analogue of the Debye–Waller factor of solids and has the effect of decreasing the measured intensity with increasing Q.

Under the assumption of a Fickian (continuous) isotropic diffusion, the scattering function corresponding to the translational motion (motion of the molecular centre of mass) has a Lorentzian shape centred at E=0, whose half width at half maximum $\Gamma(Q)$ increases with the momentum transfer according to a DQ^2 law[1]. This is expected to be a reasonable assumption as long as the molecule remains spherical and the intermolecular forces weak, which is not necessarily the case at high pressure. Literature estimations of D for room-temperature methane reached a maximum P of 0.4 GPa, at which pressure D was reported to be about 10^{-8}m^{2}s^{-1}[2]. For D=10^{-8}m^{2}s^{-1}, the $\Gamma(Q)$=$\hbar DQ^2$ relation predicts $\Gamma(Q)$~0.66 meV at Q=1 Å^{-1}, which is very compatible with our experiment. At high Q, the random-jump diffusion model suggested by Singwi and Sjölander[3] (Eq. (1) of the main text) provides a relation for the Q dependence of $\Gamma(Q)$ with only one additional parameter. Eq. (1) simplifies to $\Gamma(Q)$=$\hbar DQ^2$ for $Q\rightarrow0$ and converges progressively to $\hbar/\tau$ for $Q\rightarrow\infty$. The QENS technique provides a rather direct determination of the self-diffusion coefficient D as long as small Q values are accessible in the measurements. Conversely, the estimation of the residence time τ mainly depends on the high-Q values of $\Gamma(Q)$; its uncertainty can be significant for different reasons, such as a high instrumental background. We would like to underline that all jump diffusion models yield the same DQ^2 asymptotic behaviour at low Q (the microscopic process of Brownian jump diffusion is consistent with Fickian diffusion on a macroscopic scale).

The literature measurements of supercritical high-pressure methane at room temperature by QENS[2,4] were analysed with a roto-translational model, which assumed that the two types of motion are uncoupled. Rotational diffusion coefficients D_R ranging from 40 ps^{-1} at 0.05 GPa to 6 ps^{-1} at 0.4 GPa were reported, and their accuracy was not better than 25%[2]. 40 ps^{-1} would be too fast to be analysed by our experiment. 6 ps^{-1} corresponds to a rotational timescale of 0.0833 ps (=1/2D_R), which is just within the accessible timescales in our experiment, suggesting that the rotational motion (overall rotation of the molecule around the centre of mass) should be accessible at pressures of 0.4 GPa and above, but only on the anti-Stokes side of the spectra and at high Q. Moreover, modelling of the rotational motion can be challenging and we decided to ignore it in the present study. To limit the effect that the rotational contribution might have on our results, we excluded the points at $Q \geq 1.4$ Å^{-1} from the fits of $\Gamma(Q)$, as the intensity of the quasi-elastic terms of a rotational scattering function must tend to zero for $Q \rightarrow 0$. For example, in the model employed in ref. [2], the area of the translational component dominates over that of the rotational component at small wave vector transfers ($Q \leq 0.8$ Å^{-1}), while the opposite is true for $Q \geq 2.0$ Å^{-1}. In addition, we used a narrow fitting range close to zero energy transfer (always within –4.5 to +2 meV). The previous estimation[2] of D_R up to 0.4 GPa indicates that the quasi-elastic rotational contribution is so broad that it must be essentially seen as a flat background over this narrow region of the spectra. At high pressure, molecules penetrate deeper into the repulsive force fields of their neighbours and are likely to experience strong orientational correlations, as they also do in liquid methane at ambient pressure[5]. Orientational correlations introduce further correlations ("coupling") between the translational and rotational motions. The authors of refs. [2,4] pointed out that their model did

not perfectly reproduce the experimental spectra and that the disagreement was probably due to the neglected coupling effects between the translations and the rotations.

Supplementary Table 1. Measured pressure P, residence time τ, and self-diffusion coefficient D of methane at 300 K, with fractional uncertainty (one standard deviation) in τ and D, as well as density ρ taken from the equation of state of ref. [6] up to 1 GPa and from its extrapolation above, and shear viscosity η interpolated from refs. [7,8]. The last two columns report the factors $D\eta$ and $D\eta/\rho$, as can be calculated from the other columns of the table.

P/GPa	ρ/g cm^{-3}	τ/ps	$\delta\tau(\%)$	D/10^{-9} m^2 s^{-1}	$\delta D(\%)$	η/10^{-6} Pa s	$(D\eta)$/10^{-12} m^2 Pa	$(D\eta/\rho)$/10^{-18} m^5 Pa g^{-1}
0.12	0.3582	0.085	7	22.59	0.7	61.4	1.39	3.87
0.16	0.3849	0.087	6	18.88	0.6	74.1	1.40	3.63
0.23	0.4185	0.088	7	15.50	0.6	95.6	1.48	3.54
0.28	0.4371	0.077	5	13.68	0.3	111	1.52	3.47
0.29	0.4404	*	*	12.76	2.4	114	1.45	3.30
0.40	0.4715	0.111	4	10.77	0.4	148	1.59	3.38
0.50	0.4939	0.117	4	9.19	0.4	180	1.65	3.35
0.81	0.5451	*	*	7.08	1.9	296	2.10	3.84
1.13	0.5834	*	*	5.30	2.0	452	2.40	4.11
1.44	0.6161	*	*	3.69	3.9	637	2.35	3.82

Supplementary Note 2: Residence time

As can be seen in Supplementary Table 1, the residence time τ turned out to be small (about a tenth of a picosecond), meaning that the probed translational diffusion of methane molecules was close to being a simple Fickian diffusion. To our knowledge, no values of τ for methane are available in the literature. Typical values reported in the literature of liquid water at ambient or close-to-ambient pressure range from about 23 ps at –20°C to 3 ps at 0°C[1], to 0.4 ps at 127°C[9]. Hence, a tenth of a picosecond seems a reasonable order of magnitude for τ in methane at 300 K, which is more than 200 K above the melting temperature at ambient pressure (91 K). Moreover, it seems reasonable to expect smaller values of the residence time in methane compared to an associated (hydrogen-bonded) liquid like water.

The statistics of the data taken in the Paris–Edinburgh press is not sufficient for a precise determination of τ and Eq. (1) of the main text provides τ values affected by extremely large uncertainties ranging from 34 to 430%, which are omitted in Supplementary Table 1. For these pressure points, the $\Gamma(Q)$=$\hbar DQ^2$ relation can be used instead of Eq. (1) and the obtained values of D are identical within the error bars. For the points taken in the gas pressure cell up to 0.5 GPa, τ is affected by an uncertainty of typically 5% and shows a moderate increasing trend with increasing P, as reported in Supplementary Table 1.

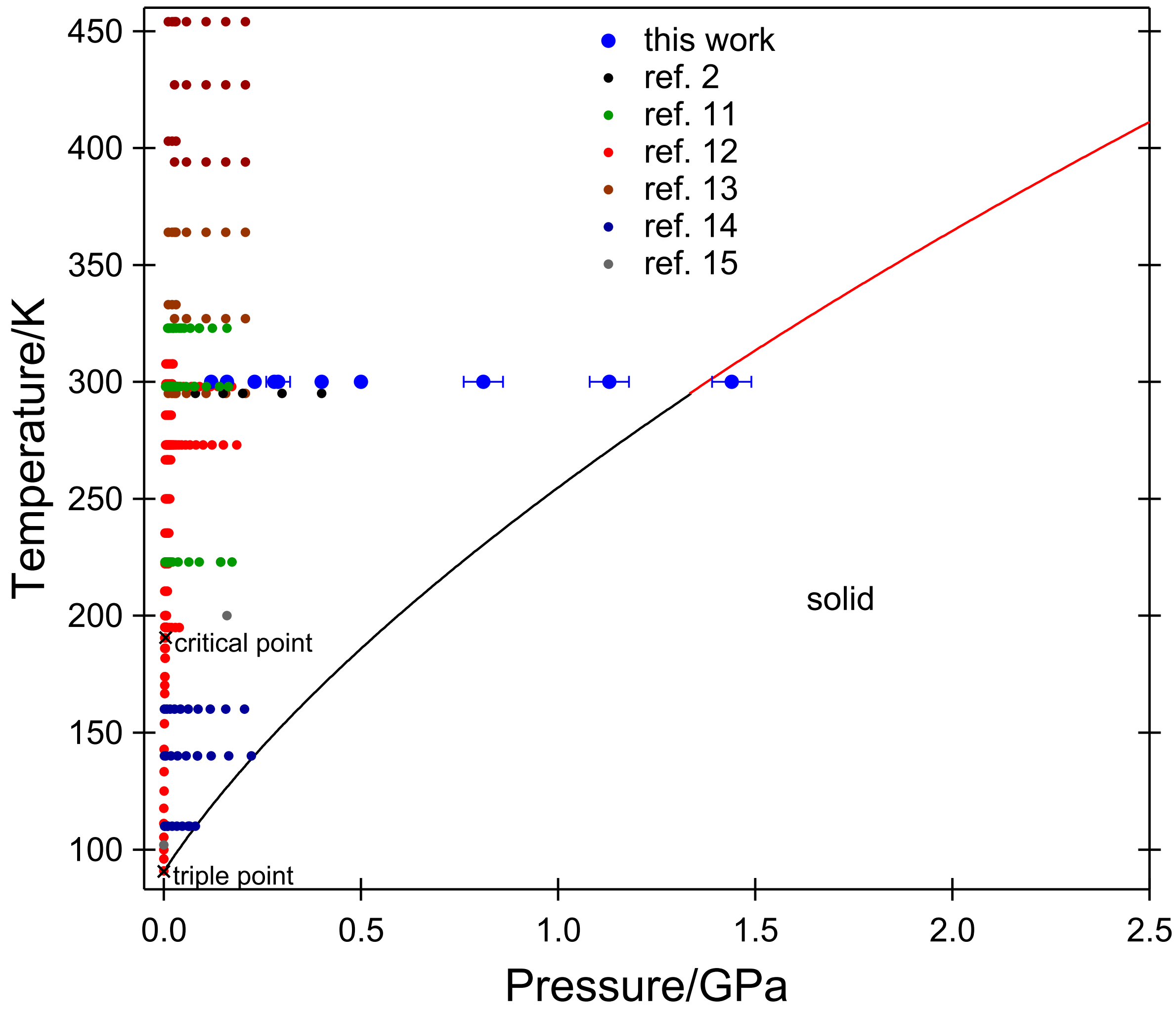

Supplementary Figure 1. Phase diagram of methane. T-P phase diagram of methane with the low-T melting line (black line) from ref. [6] and the high-T melting line (red line) from ref. [10]. In addition to the room-temperature measurements commented in the main text, Harris[11] measured D along the isotherms 223 and 323 K up to 0.17 and 0.16 GPa, respectively. Oosting and Trappeniers[12] measured D along the isotherms 195 and 273 K up to 0.04 and 0.19 GPa, respectively, along the liquid-vapor coexistence line between the triple point and 186 K, and along five isochores at temperatures from 170 to 308 K and up to a maximum pressure of 0.02 GPa. Greiner-Schmid and co-workers[13] measured D along seven isotherms between 327 and 454 K up to maximum pressures of 0.207 GPa. Harris and Trappeniers[14] measured D along the isotherms 110, 140, and 160 K up to 0.08, 0.22, and 0.20 GPa, respectively. Johnson and Olsson[15] measured D at 102 K and 1 bar, and 200 K and 0.16 GPa. Horizontal error bars show the uncertainties in pressure.

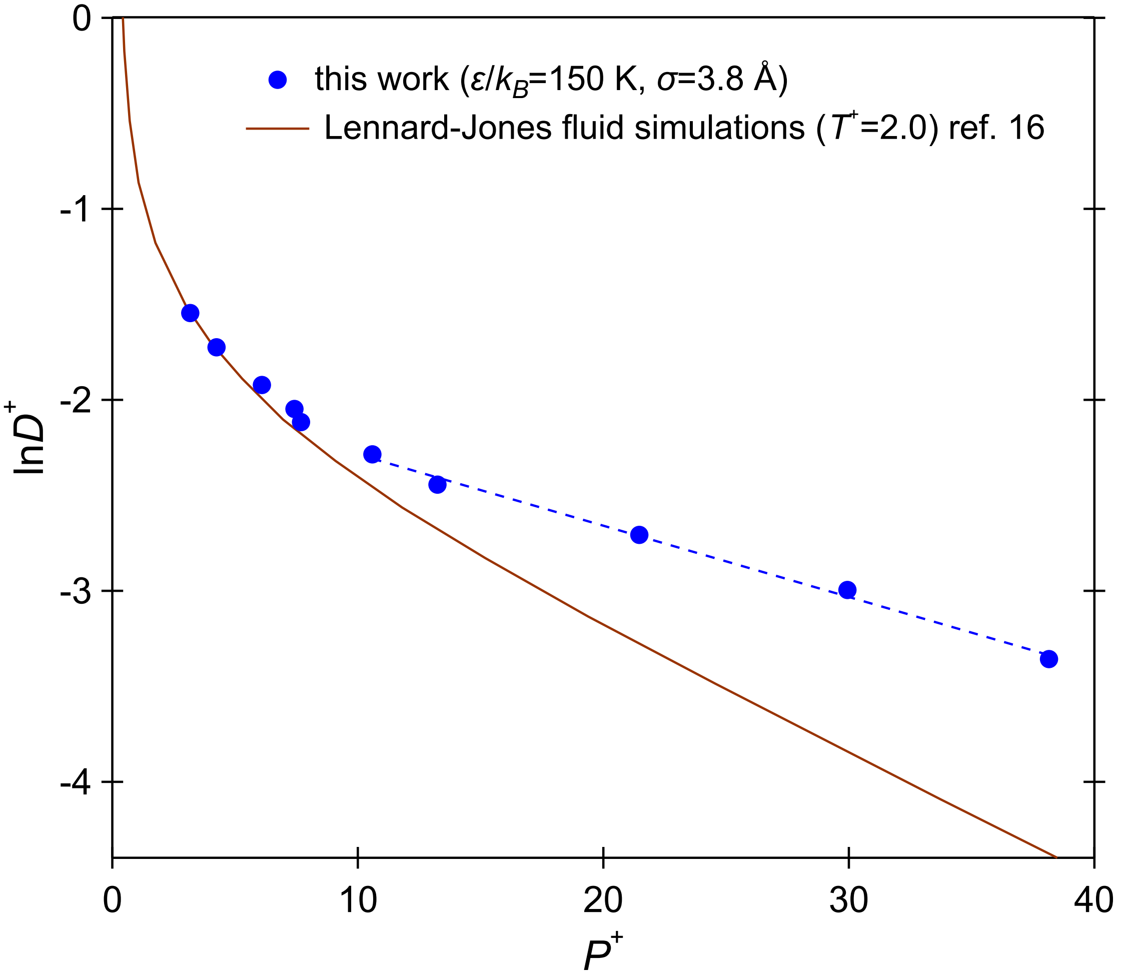

Supplementary Figure 2. Lennard–Jones model. Logarithm of the dimensionless reduced self-diffusion coefficient D^{+}=$D\sqrt{m/\epsilon\sigma^2}$ as a function of dimensionless reduced pressure P^{+}=$P\sigma^3/\epsilon$ from our data (the employed values of ϵ and σ are indicated in the legend) and from the results of the molecular dynamics simulations of the Lennard–Jones fluid reported in ref. [16] for T^{+}=$k_{\mathrm{B}}T/\epsilon$=2.0. It can be seen that a linear dependence describes our data at high pressure fairly well, but the slope is very different from that predicted for the Lennard–Jones fluid.

Supplementary Note 3: Reduced self-diffusion coefficient D^*

The dimensionless reduced self-diffusion coefficient D^* defined by Dymond[17] is given by

$$D^* = \frac{nD}{(nD)_0}\left[\frac{V}{V_0}\right]^{2/3}, \tag{1}$$

where $(nD)_0$ is the value of nD for the dilute gas, V is the molar volume and V_0 the volume of close packing:

$$V_0 = \frac{N_\mathrm{A}\sigma^3}{\sqrt{2}}. \tag{2}$$

For hard spheres, $(nD)_0$ is given by the gas kinetic Chapman-Enskog theory:

$$(nD)_0 = \frac{3}{8\sigma^2}\left(\frac{RT}{\pi M}\right)^{1/2}. \tag{3}$$

Using Eqs. (2) and (3), Eq. (1) can be written as

$$D^* = \frac{8}{3}D\left(\frac{2N_\mathrm{A}}{V}\right)^{1/3}\left(\frac{\pi M}{RT}\right)^{1/2}. \tag{4}$$

Finally, Eq. (4) is equivalent to

$$D^* = 17.44\times 10^7\frac{D}{V^{1/3}}\left(\frac{M}{T}\right)^{1/2}, \tag{5}$$

if the unit of D is $\mathrm{m}^2\ \mathrm{s}^{-1}$, the unit of V is $\mathrm{m}^3\ \mathrm{mol}^{-1}$, the unit of M is kg mol^{-1}, and the unit of T is K. We calculated D^* for our experimental values of D, V=M/ρ, M=16.04 g mol^{-1} and T=300 K. The result is plotted in Supplementary Fig. 3 as a function of reduced number density $n\sigma^3$, where n=$\rho N_\mathrm{A}/M$ and σ was chosen to be 3.6 Å. Similarly, we calculated D^* and $n\sigma^3$ for literature data[11,14] along six other isotherms reaching a maximum pressure of 0.22 GPa, using the

values of σ indicated in the legend of Supplementary Fig. 3. This set of six values was found in ref. [14] so that the reduced diffusivity isotherms fall on a common curve when plotted against reduced number density. It can be seen from Supplementary Fig. 3 that the six literature isotherms do fall on a common curve while our isotherm deviates significantly. Supplementary Figure 3 also reports D^* as a function of $n\sigma^3$ for the smooth hard-sphere fluid as obtained computationally in ref. [18]. Supplementary Figure 3 is another way of visualizing the finding presented in Fig. 4a of the main text: There is no constant sphere diameter σ for which our experimental results can be matched with the expectation for a smooth hard-sphere fluid. It is also interesting to note that the wide pressure range explored in the current study allows us to see a considerably larger deviation from the smooth hard-sphere behaviour than that observed in liquid methane for $n\sigma^3$>0.86 in ref. [14]. Supplementary Figure 3 also shows that there is a clear deviation of the literature results[11,14] from the smooth hard-sphere behaviour for $n\sigma^3$ below $\sim$0.6.

The reduced number density corresponding to freezing of the hard sphere fluid is 0.9392[18] but ref. [18] also reported results in the metastable fluid region, up to $n\sigma^3$=1.01. Our values of D^* at the two highest investigated pressures can be superposed to the full line of Supplementary Fig. 3 for $\sigma\sim$3.43 Å. The resulting $n\sigma^3$ is about 0.93 at our highest pressure point. On the other hand, from the equation $n\sigma^3$=0.9392 and the density of fluid methane at the freezing pressure, it is possible to independently calculate the equivalent diameter at a certain temperature by assuming that methane is a close packed hard sphere fluid. At 1.38 GPa and 300 K, ρ=0.6097 g cm^{-3} therefore n=0.02289 Å^{-3} and one finds σ=3.449 Å. This is in fairly good agreement with the value

mentioned above (3.43 Å) or the (identical) one that could be deduced from Fig. 4a, showing the essential correctness of our experimental results. It must be stressed that the density value at 1.38 GPa used here had to be extrapolated from the literature data[6] existing up to 1 GPa. In figure 5 of ref. [14], Harris and Trappeniers showed that σ values from i) the comparison of the experimental diffusion coefficient with that of the smooth hard-sphere fluid at moderate pressures and ii) the experimental density of fluid methane at freezing coincide at low temperature. However, the two approaches provide progressively diverging diameters above $\sim$170 K[14]. One can then infer that the equivalent hard-sphere diameter of methane must be reduced upon compression along isotherms for T>170 K, but entire isotherms have to be measured for this effect to be visible (pressures comparable to the freezing points have to be achieved).

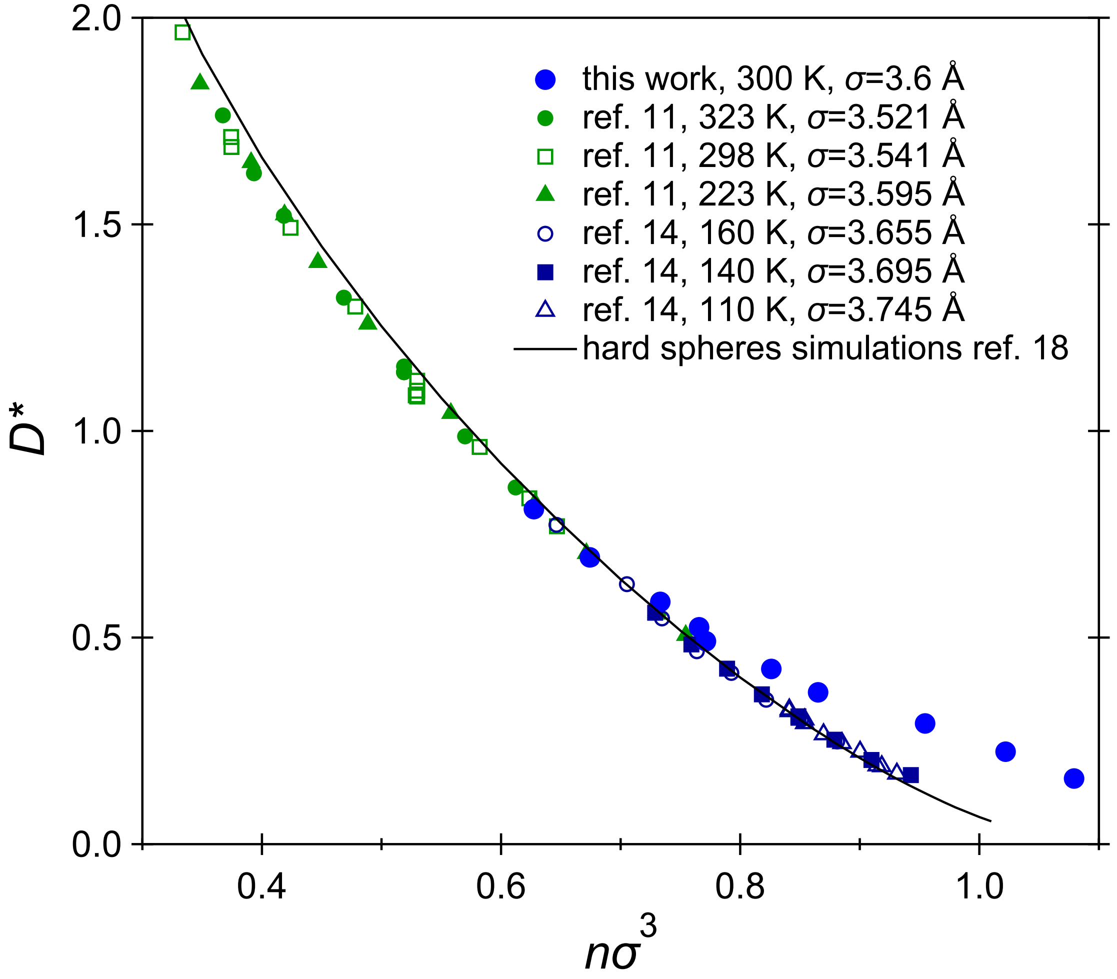

Supplementary Figure 3. Reduced diffusion coefficient and hard-sphere model. Reduced self-diffusion coefficient of methane from our experimental data along the 300 K isotherm and from the experimental data of refs. [11,14] along other isotherms, as a function of reduced number density $n\sigma^3$. The (ρ-independent) σ values employed for each isotherm of this plot are indicated in the legend. The solid line is the σ- and T-independent result obtained from the molecular dynamics simulations of the smooth hard-sphere fluid reported in ref. [18].

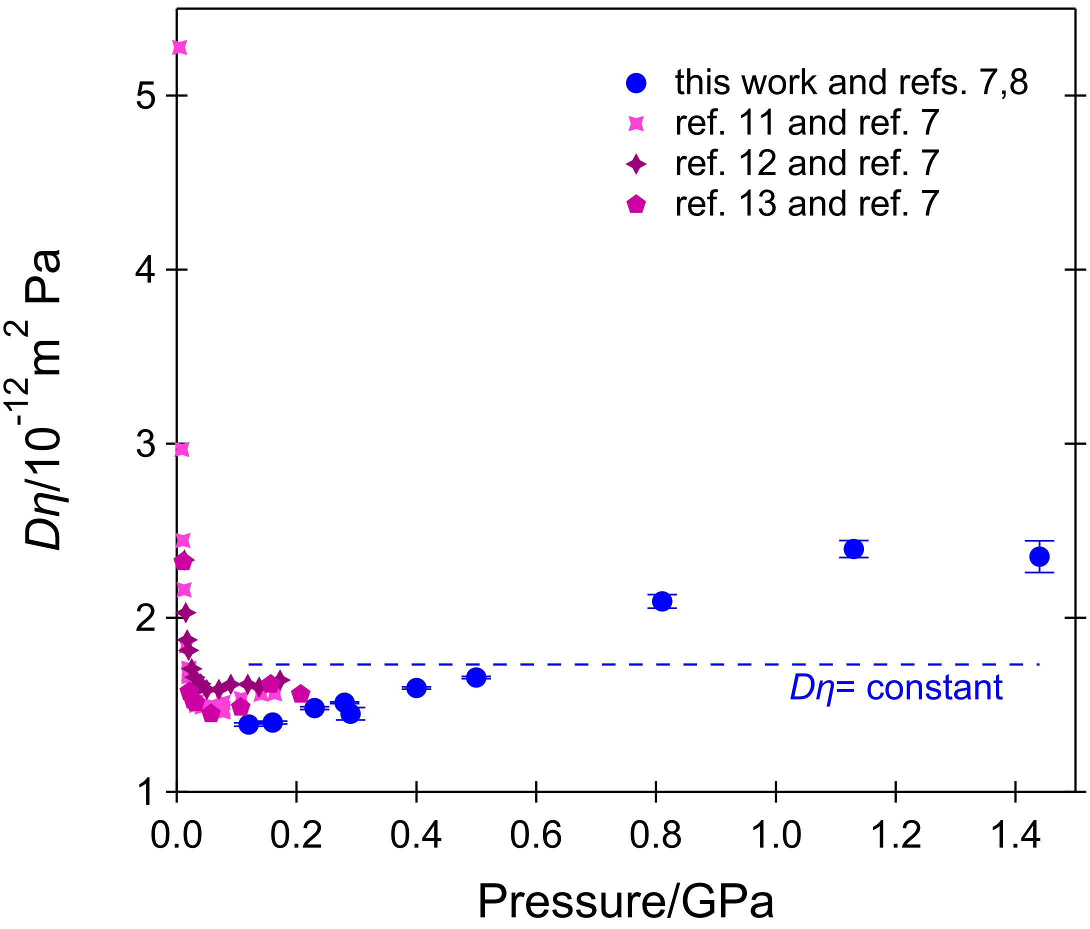

Supplementary Figure 4. Test of the Stokes–Einstein–Sutherland relation at room temperature. Pressure dependence of the product between the self-diffusion coefficient D and the viscosity η, calculated from the D values of this work and η values at room temperature interpolated from refs. [7,8], and from the room-temperature D values of refs. [11–13] and η values interpolated from ref. [7]. The error bars were obtained by propagating the errors in D only. The SES relation predicts a constant product $D\eta$.

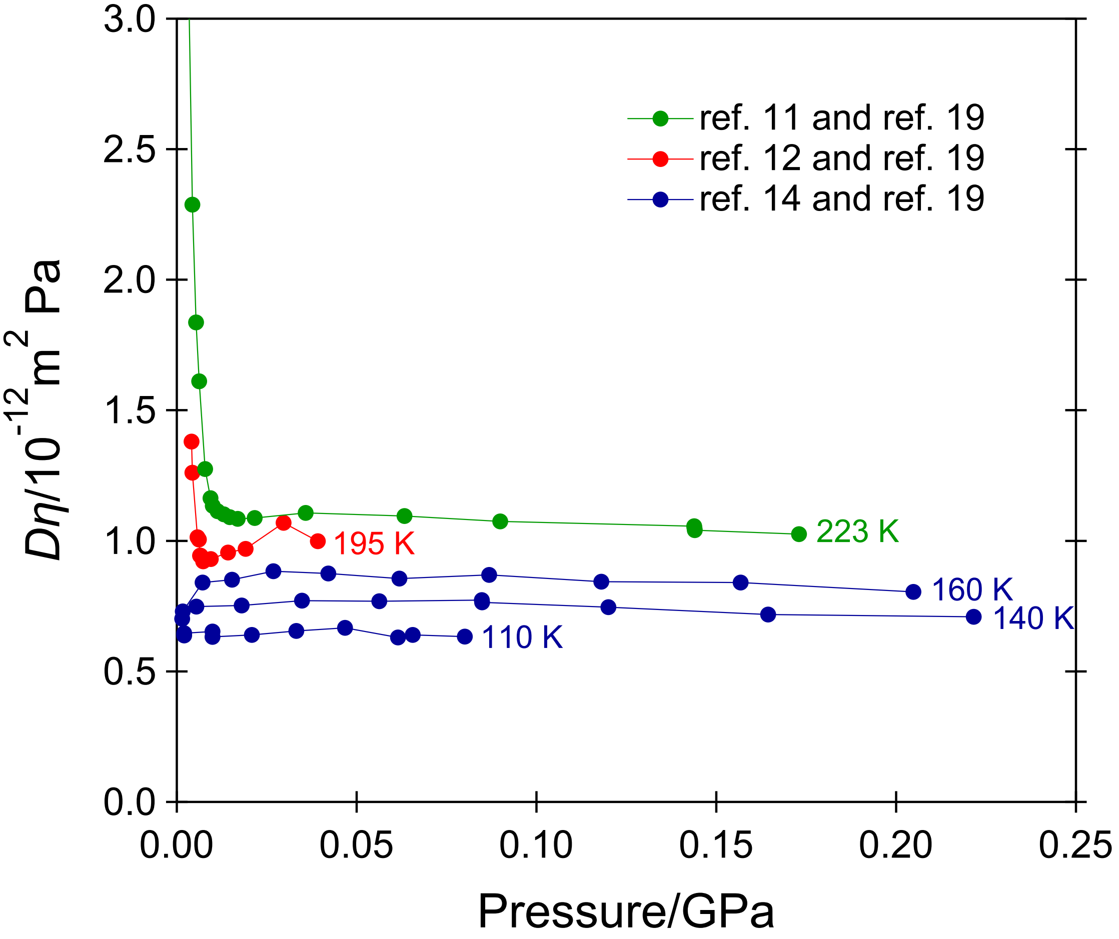

Supplementary Figure 5. Test of the Stokes–Einstein–Sutherland relation at low temperature. Pressure dependence of the product between the self-diffusion coefficient D and the viscosity η along some low-temperature isotherms from literature data. D values are experimental values from refs. [11,12,14] and η values are predicted values from ref. [19].

Supplementary Note 4: Diameters and C values derived from the Stokes–Einstein–Sutherland equation

At the lowest pressures of our experiment, $D\eta$ is about 1.4×10^{-12}m^2 Pa (see Supplementary Fig. 4) and the Stokes–Einstein–Sutherland relation (Eq. (4) of the main text) is satisfied with an effective molecular diameter σ=4.7 Å for C=2 (slip limit), or σ=3.2 Å for C=3 (stick limit). This low-P value of the SES relation could be used to extrapolate D to higher pressures; one would find 2.2×10^{-9}m^{2}s^{-1} at 1.44 GPa, for example, which is certainly not a satisfactory prediction when compared to our experimental results. At the highest investigated pressures, $D\eta$ is about 2.4×10^{-12}m^2 Pa (see Supplementary Fig. 4) and Eq. (4) is satisfied with σ=2.8 Å for C=2, or σ=1.9 Å for C=3. This means that Eq. (4) cannot be satisfied over the entire investigated P range for a unique diameter σ even if C is allowed to vary from 3 to 2. It can be satisfied at all pressures if σ is allowed to change for a given C. However, an unrealistically large variation would be needed, for example from 3.8 to 2.2 Å for C=2.5. These effective diameter values may be compared with the van der Waals diameter of 3.78 Å for methane[20].

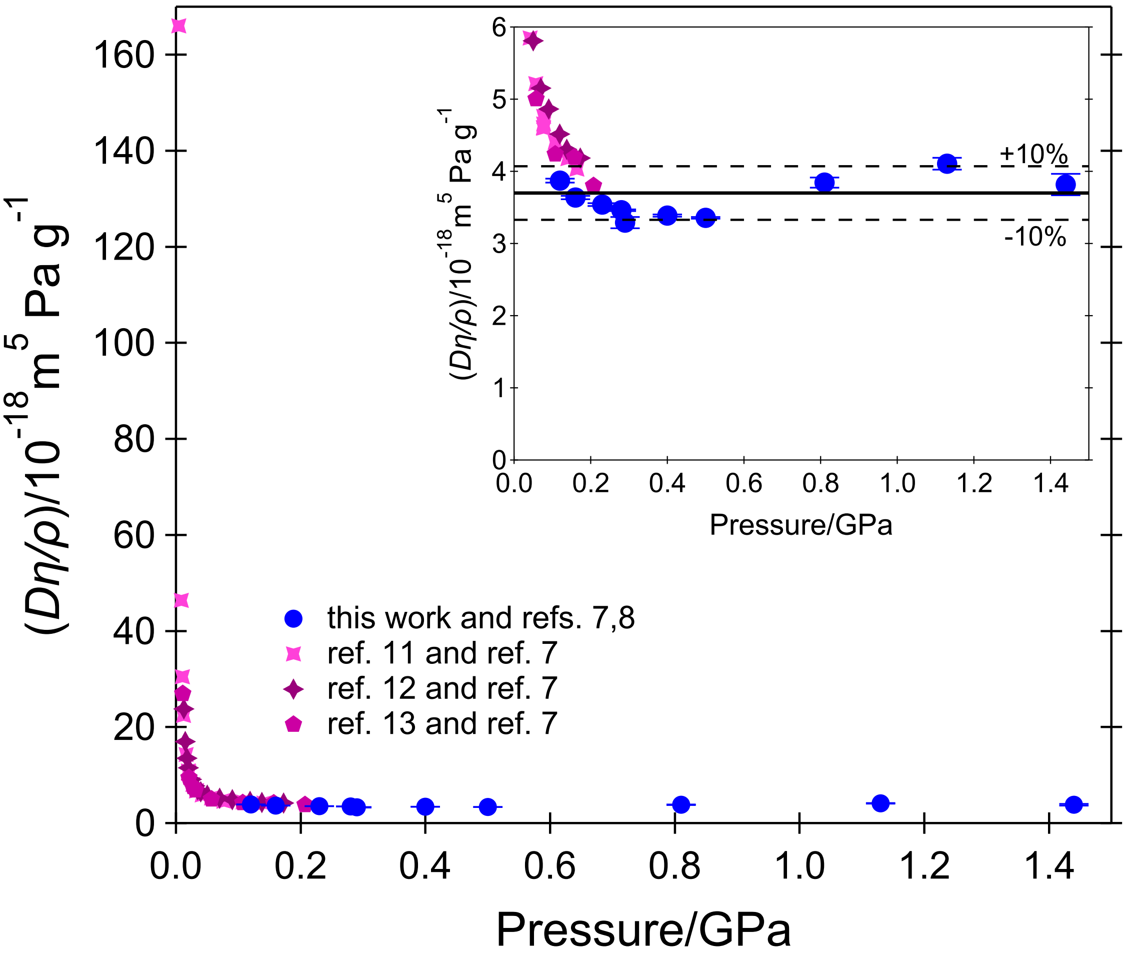

Supplementary Figure 6. Test of the $D\eta/\rho$=constant relation at room temperature. Pressure dependence of the factor $D\eta/\rho$ at room temperature, calculated from the D values of this work and of refs. [11–13], and η values interpolated from refs. [7,8]. In the inset, the same plot is zoomed over a shorter y-axis range for better visibility. The error bars were obtained by propagating the errors in D only. $D\eta/\rho$ is found to be constant within ±10% in the pressure range of this work.

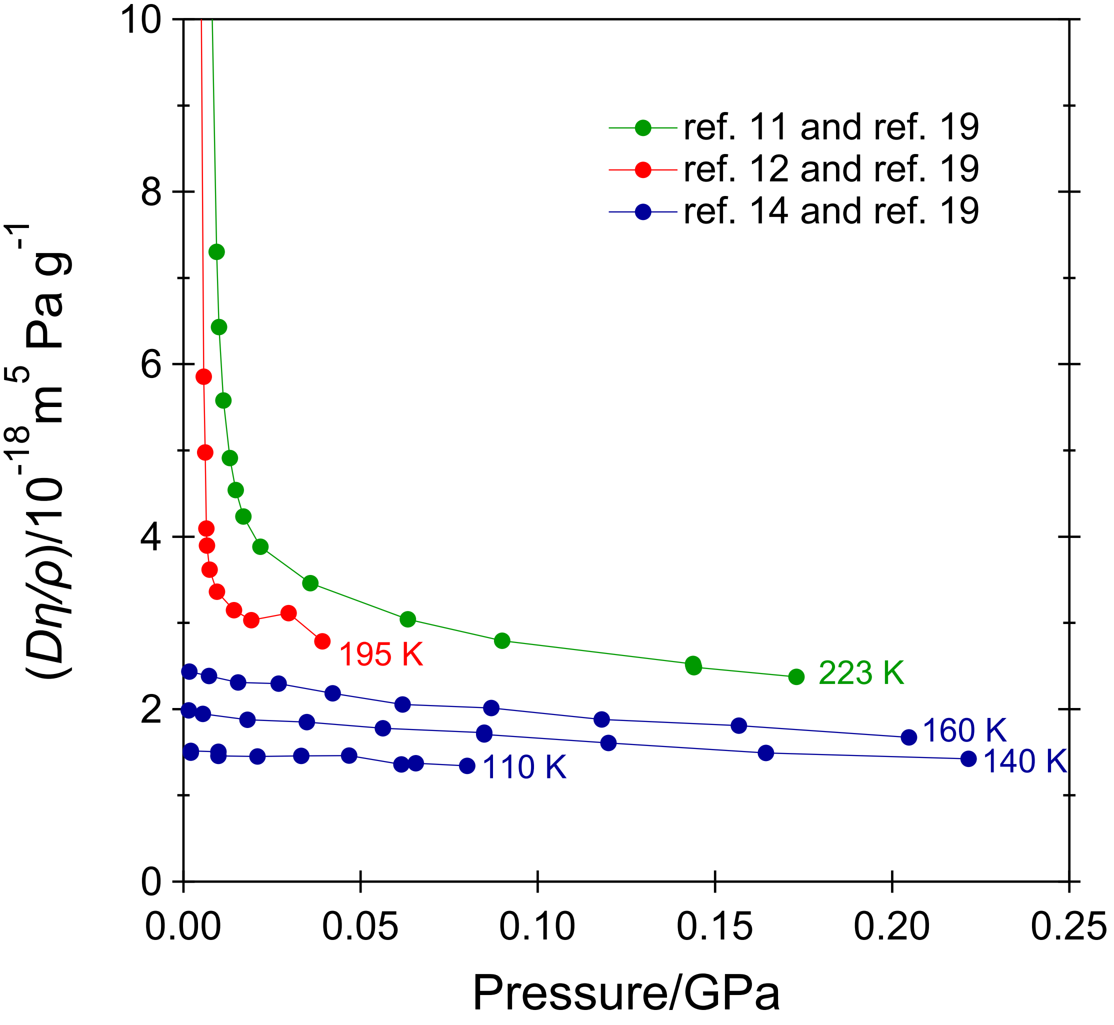

Supplementary Figure 7. Test of the $D\eta/\rho$=constant relation at low temperature. Pressure dependence of the factor $D\eta/\rho$ along some low-temperature isotherms from literature data. D values are experimental values from refs. [11,12,14] and η values are predicted values from ref. [19].

Supplementary References

1. Teixeira, J., Bellissent-Funel, M. C., Chen, S. H. & Dianoux, A. J. Experimental determination of the nature of diffusive motions of water molecules at low temperatures. *Phys. Rev. A* **31**, 1913–1917 (1985).

2. Johnson, T. & Olsson, L. G. The molecular dynamics in highly compressed methane gas studied by slow neutron scattering experiments. *Physica* **115B**, 15–26 (1982).

3. Singwi, K. S. & Sjölander, A. Diffusive motions in water and cold neutron scattering. *Phys. Rev.* **119**, 863 (1960).

4. Johnson, T. & Olsson, L. G. On the dynamics in compressed methane gas at 3 kbar by inelastic neutron scattering. *J. Phys. B: At. Mol. Phys.* **16**, 1853–1861 (1983).

5. Sampoli, M., Guarini, E., Bafile, U. & Barocchi, F. Orientational and translational correlations of liquid methane over the nanometer-picosecond scales by molecular dynamics simulation and inelastic neutron scattering. *J. Chem. Phys.* **135**, 154508 (2011).

6. Setzmann, U. & Wagner, W. A new equation of state and tables of thermodynamic properties for methane covering the range from the melting line to 625 K at pressures up to 1000 MPa. *J. Phys. Chem. Ref. Data* **20**, 1061–1151 (1991).

7. Van der Gulik, P. S., Mostert, R. & Van den Berg, H. R. The viscosity of methane at 25°C up to 10 kbar. *Physica A* **151**, 153–166 (1988).

8. Abramson, E. H. Viscosity of methane to 6 GPa and 673 K. *Phys. Rev. E* **84**, 062201 (2011).

9. Bove, L. E. *et al.* Translational and rotational diffusion in water in the Gigapascal range. *Phys. Rev. Lett.* **111**, 185901 (2013).

10. Abramson, E. H. Melting curves of argon and methane. *High Press. Res.* **31**, 549–554 (2011).

11. Harris, K. R. The density dependence of the self-diffusion coefficient of methane at –50°, 25° and 50°C. *Physica* **94A**, 448–464 (1978).

12. Oosting, P. H. & Trappeniers, N. J. Proton-spin–lattice relaxation and self-diffusion in methanes: IV. Self-diffusion in methane. *Physica* **51**, 418–431 (1971).

13. Greiner-Schmid, A., Wappmann, S., Has, M. & Lüdemann, H.-D. Self-diffusion in the compressed fluid lower alkanes: Methane, ethane, and propane. *J. Chem. Phys.* **94**, 5643 (1991).

14. Harris, K. R. & Trappeniers, N. J. The density dependence of the self-diffusion coefficient of liquid methane. *Physica* **104A**, 262–280 (1980).

15. Johnson, T. & Olsson, L. G. The temperature dependence of the molecular dynamics in methane in high density. *Physica* **122B**, 227–235 (1983).

16. Baidakov, V. G., Protsenko, S. P. & Kozlova, Z. R. The self-diffusion coefficient in stable and metastable states of the Lennard–Jones fluid. *Fluid Ph. Equilibria* **305**, 106–113 (2011).

17. Dymond, J. H. The interpretation of transport coefficients on the basis of the Van der Waals model: I dense fluids. *Physica* **75**, 100–114 (1974).

18. Pieprzyk, S., Bannerman, M. N., Brańka, A. C., Chudak, M. & Heyes, D. M. Thermodynamic and dynamical properties of the hard sphere system revisited by molecular dynamics simulation. *Phys. Chem. Chem. Phys.* **21**, 6886–6899 (2019).

19. Younglove, B. A. & Ely, J. F. Thermophysical properties of fluids. II. Methane, ethane, propane, isobutane, and normal butane. *J. Phys. Chem. Ref. Data* **16**, 577 (1987).

20. Edward, J. T. Molecular volumes and the Stokes-Einstein equation. *J. Chem. Educ.* **47**, 261 (1970).
